# College Students' Perception of Snacks Sold in Vending Machines in the US: A Mixed-Methods Study

**DOI:** 10.3389/fnut.2021.742121

**Published:** 2021-10-27

**Authors:** Cristiana Assumpção Mengarelli, Christie Kirchoff, Cristina Palacios

**Affiliations:** ^1^Department of Dietetics and Nutrition, Robert Stempel College of Public Health & Social Work, Florida International University, Miami, FL, United States; ^2^Department of Health Promotion & Disease Prevention, Robert Stempel College of Public Health & Social Work, Florida International University, Miami, FL, United States

**Keywords:** college students, vending machines, snacks, mixed-methods study, Hispanic serving institutions, perception

## Abstract

**Introduction:** Food in vending machines in US colleges contain limited nutritious foods available for purchase, which could affect the food choices made by students leading to poor diet quality. Interventions to improve college foodscapes usually follow a top-down approach and fail to affect dietary behavioral changes ultimately. This research aims to uncover what students want and ways to achieve change.

**Methods:** The mixed-methods approach included peer-led qualitative focus group discussions and a brief quantitative questionnaire on satisfaction from foods available in vending machines. A convenience sample of 20 students (15 females) was recruited from a Hispanic serving institution for this study.

**Results:** Vending machines were perceived as convenient, plentiful, and unhealthy. Students expressed dissatisfaction with both the variety and nutritional quality of snacks in vending machines. Suggestions for improvement included more fresh items (fruits and vegetables) and refrigerated items with higher protein content (low-fat yogurt, hummus, and peanut butter). To implement these improvements, participants discussed the cost and feasibility of perishable items. Increasing awareness and partnering strategies were proposed to mediate potential cost and buy-in obstacles as was elevating the appeal of healthy vending machines with technological enhancements that draw customers in and educate.

**Conclusion and Implication for Practice:** This group of college students was eager for positive changes in foods sold in vending machines and understand the major difficulties. The suggested changes may help this and other colleges develop policies to regulate the foods in vending machines to promote overall health and help prevent chronic diseases in the future.

## Introduction

In the United States (US), pre-packaged processed food items have grown in popularity over the years. Pre-packaged processed foods, also commonly known as snacks, are notoriously low in nutrient content and high in sugar, sodium, and fats (1–7). Due to their longer shelf life, they have become a mainstay in vending machines (VM) and other fast-paced food environments (4, 8–10). These items are particularly popular in educational institutions in the US and most snacks sold in VM are of low nutritional value foods, such as soft drinks, chips, and sweets (4, 8, 9, 11).

Studies among college campuses have shown that unhealthy on-campus food choices are one of the many environmental pillars that may increase the probability of weight gain and waist circumference (12–14), which contribute significantly to chronic disease risk factors (15). The use of VM on college campuses has also been shown to influence dietary intake and diet quality (8) and it has been shown that college students tend to choose options with a less nutritional value over healthier snacks when purchasing from VM (16). This is important as during the college years, young adults make dietary decisions that would likely be maintained throughout the rest of their lives, influencing their future health status (17).

VM food purchasing is influenced by environmental cues (18, 19), social influences, lack of time, physical and financial accessibility, and convenience (10, 16, 17). Increasing knowledge on healthful food options usually helps individuals make healthier choices over a poorer alternative (20, 21). However, barriers are more powerful than enablers (22), as most VM do not offer healthy alternatives, impeding students from choosing healthier options. Therefore, availability and accessibility to healthy foods, along with information on which foods are healthier, are the most critical factors that determine students' daily food options (11, 23).

Gathering preliminary information on food choices and perceptions on college campuses is paramount to understand how to implement interventions to improve VM food options. Intervention studies have attempted to manipulate pricing, using the traffic light system, providing nutritional information, increasing availability of healthy items, and other interventions (24–34), but the results from these studies have not been consistent in affecting purchasing behaviors. Additionally, there are very limited studies focusing on students' attitudes, higher education institutions, and among Hispanics, a group with a high risk of chronic conditions (35–39). The student frame of reference is essential to understand and adapt culturally appropriate interventions to improve VM food products at Hispanic serving institutions. Therefore, this research aimed to discover the student viewpoint of VM and how VM might be improved. While prior research has explored improving the VM environment through top-down approaches, the current study posits that student input is a critical component of intervention success.

## Methods

### Setting

This mixed-methods study focused on the perceptions of college students on VM food availability and preferences at the main Florida International University (FIU) campus, a Hispanic serving institution, and considered their opinions on how to improve the food choices in these dispensers. Student enrollment at this campus was 58,711 in 2019, of which 67% were Hispanic (40). Classes are scheduled from 7:00 AM to 10:30 PM, therefore, some students stay on campus until very late; most dining locations close between 5:00 and 8:00 PM. In fact, the libraries are open from 6:00 AM to 10:00 PM and there are several VM inside the library. In total, our research team has counted over 200 VM throughout the main campus.

This study used the Ecological framework as the framework to explain some of the higher-level influences of an individuals' choices and perceptions of VM foods. The ecological framework helps to explain some of the higher-level influences on dietary choices (41). The framework consists of macro-level and physical environments and societal and individual factors. The macro-level effects are societal norms, industry standards, marketing, and governmental policies that influence trends in food supplies and products. The physical environment is associated with the places where we spend a large portion of our time such as work, schools, homes, and neighborhoods, and exert influence through the number and type of convenience stores, VM, restaurants, and products available in those spaces. The social factors are family friends and peers. Finally, the individual elements are knowledge, attitudes, beliefs, practices, demographic characteristics, preferences, motivation, self-efficacy, values, skills, and lifestyle. The Ecological framework was used to guide the creation of the qualitative questionnaire to ensure it covered all four levels of influence.

This manuscript was prepared following the standard guidelines for mixed-methods studies (42).

### Population Sample

The initial sample was designed to be 20 participants or until the data gathered from quotes became saturated. College students were recruited through flyers posted in different places on campus and by a mass e-mail sent to FIU professors asking them to share with their students, with pertinent contact information for interested participants to sign up. Those interested were provided with the dates, times, and locations of the focus groups, via email. The inclusion criterion for population selection was being a registered student 18 years of age or older. The exclusion factors were professors, visitors, and university staff.

Participants were compensated with a USD 20.00 gift card after completing the session. Healthy snacks and beverages were also provided. Data was collected during the Summer and Fall of 2018. The study was approved by the FIU Institutional Review Board and students provided a written consent and media release form before participating in the study.

### Data Sources and Measurements

#### Focus Groups

The focus group interview guide was developed based on a thorough literature review guided by the factors considered in the ecological framework as described above. It specifically aimed to understand students' perceptions of VM and VM content, identify if students were satisfied with the VM environment, if they wanted to see improvements, and how they envisioned that change. The finalized guide was reviewed by two experts. Recruited students were asked to choose one of three focus groups to participate in based on their schedule. Each focus group was limited to 10 students to allow all students to voice their opinions. All focus groups were moderated by the same peer graduate student (CAM) who received intensive training on how to conduct focus groups. Another graduate student helped with the logistics of all focus groups, such as providing copies of the questionnaire, making sure the session was recorded and providing payment to each participant at the end. These focus groups were anonymous and audio-recorded and lasted about 40 min. Students were identified by number and were asked to verbalize their identification number before voicing their opinions. Focus group discussions were conducted until saturation was reached.

#### Questionnaire

Participants were also asked to complete a brief questionnaire that included two sections: demographics and food preferences from VM. For demographics, there were a total of six questions, such as age, gender, major, educational level, and food preferences in terms of cultural influence. For food preferences from VM, there were five questions intended to help triangulate focus group findings such as open-ended questions about the VM food products they consumed and their satisfaction with the products currently offered in VMs; the frequency of VM use, satisfaction with the selection, satisfaction with the nutrition content of foods in VM, and reasons for selecting the foods purchased in VM. These were rated using a 5-point Likert scale. Questions used in this survey were based on another questionnaire previously used in other studies to explore VM use and attitudes toward healthier snack products and price (43). It was adjusted for the current study to include demographic information, the specific food culture that they most related to, the frequency with which they purchased from VM, and their level of satisfaction with the current food items sold in these dispensers.

### Statistical Plan

#### Quantitative

For descriptive analysis, we used frequencies for categorical variables (gender, education level, major, food culture, dietary restrictions or preferences, and satisfaction degree with selection and nutritional quality of VM foods offered), and median (25th, 75th percentiles) for the continuous variable (age). The data was entered and analyzed in Microsoft Excel (Microsoft Corporation, Redmond, WA).

#### Qualitative

Answers from focus groups were analyzed by transcribing all the comments reported by participants, verbatim. Open coding was conducted by one researcher (CAM) in discussion with a second researcher (CP) using NVIVO software, which aided in identifying axial codes. Codes were further analyzed by two independent researchers (CAM, CK) and organized into themes. The analysis was iterative, and researchers consulted and compared the data to itself repeatedly to arrive at the themes generated from it.

Data and software code is available upon request.

## Results

A total of 45 showed interest in participating in the study; the first 20 students that were able to accommodate the focus group dates in their schedules were those that attended and participated. A total of seven students participated in the first focus group, four in the second focus group, and nine in the last focus group.

### Quantitative

The diverse sample of participants was between 18 and 38 years old seeking an undergraduate (55%) or graduate degree (45%) in various majors, such as health-related majors (nursing, public health, and dietetics and nutrition), business, architecture, languages, social work, sociology, and education. Table 1 illustrates the demographic characteristics of the sample.

**Table 1:** Demographic characteristics of study participants (*n* = 20).

**Variable**	**N (%) or median (25th, 75th)**
Age (years), Median (25th, 75th)	24 (22,27)
Females, % (n)	15 (75)
**Degree level, %**	
Undergraduate	11 (55)
Graduate	8 (40)
**Food culture, % (** ***n*** **)**	
Latin-American/Hispanic/ Caribbean	8 (62)
African American/North American	2 (15)
Mediterranean	1 (8)
Asian	0 (0)
Vegan/vegetarian	1 (8)

A total of 85% of the participants reported using VMs on campus. The items frequently purchased by participants were salty snacks (39%), sweets and candy (45%), and cereal and granola bars (11%). Fifty-five percent reported being unsatisfied with the variety and 95% with the nutritional content of VM food products offered on campus. VM use and satisfaction with VM content are presented in Table 2. Females reported more frequent VM use than males with 80% using them at least a few times per month compared to 40% of males. Dissatisfaction with VM selection increased with the frequency of VM use whereas dissatisfaction with the nutritional content of snack foods remained the same despite the frequency of use.

**Table 2:** Vending machine purchasing behaviors.

**Variable**	***N* (%)**
**Frequency of purchasing in VMs**	
Every day/most days	2 (10)
At least once weekly	3 (15)
Few times per month	9 (45)
Once per month or less	3 (15)
Never	3 (15)
**Food selection in VMs**	
Not satisfied/somewhat unsatisfied	11 (55)
Neutral	4 (20)
Somewhat satisfied/satisfied	5 (25)
**Nutritional value of foods in VMs**	
Not satisfied/somewhat unsatisfied	19 (95)
Neutral	1 (5)
Somewhat satisfied/satisfied	0 (0)

### Qualitative

The focus group discussions revealed three main themes that represent students' perceptions of VM and how to improve them. The themes labeled “VMs are convenient”, “VMs are in need of improvement”, “improving VM content will be challenging” are described in depth below and illustrative quotes are presented in Table 3.

**Table 3:** Selected quotes illustrating themes and subthemes.

**Theme**	**Subtheme**	**Exemplifying quotation**
VMs are convenient	Accessible	“They're basically 24 h, and there's a lot of them on campus, so they're easy to access, especially here on campus really late or really early in the morning.” “There is one in every corner, so it's easy to get to them and fast.” “The fact that you could pay however you want, ApplePay, card, cash, coins, that makes it easy.”
	Cheap	“I also really agree with convenience, like if you just want to eat something quick on the go, but also one of the best things of the vending machines is that it's cheap. Mostly cause you don't want to eat something light in a restaurant or something, you just want to spend a dollar or two dollars at most just to like quickly satisfy your hunger for just the time being. Until your able to have a meal at your house or something. But yeah, it's like on-the-go food and it's the best thing.”
VMs need improvement	Unhealthy	“I also think that they're too unhealthy like there might be. I think I've seen a couple of VM that they're like, specifically for healthy foods, they have nuts, they have fruit packets, dried fruit, but you know, generally, most of them are just like chocolate bars, potato chips, and all that candy, unhealthy stuff.” “All options are junk food. I wish they would sell healthier options.”
	Lack of Variety	“Not a larger variety available.” “Selection and quality are low for the price point.”
Improving VM will be challenging	Education	“I'm pretty sure if we're talking fresh foods in VM they're gonna have to be changed often and education I guess in a way because I would pay more at a VM for an apple than I would for chips. So, it's just understanding that although we're gonna get healthier alternatives, there may be a higher cost to the consumer for that, you know, um, so people kind of understand. Low cost would be great, but I guess if it means cheaper and unhealthy or a little bit pricey and healthy, I'd go with the pricier and healthier.” “That also goes back to knowing that this kind of food has this much protein, this much fiber if people don't know they're not going to get that option. They'd rather get something that looks like it's a lot more food than getting. Oh, this might be smaller, but it actually has a lot more protein. It goes back to nutrition education.”
	Shelf Life and Price	“Anything that's going to be preserved usually is made up of bad ingredients, not nutrient-dense or healthy ingredients for you, so although they may have healthier options, still it's something that can sit there, have a long shelf life, and things that have long shelf lives are not really the best option for you, nutritionally.” “I think that maybe an issue with having healthy VM is probably a lot of the food is highly processed, and it might be hard to maintain, like the longevity of the food in the VM.”
	Funding	“We need funding to get started. It all depends on where we get it from, and how we push an initiative to actually jumpstart this whole thing, so that umm, you know, these vending machines will start to become a replacement and healthier alternatives.”
	Consensus building	“They maybe have to put in a test machine in place” “You can test it out by putting it next to a regular vending machine. The healthy one next to the regular one and test it that way. What do people want?” “So I don't know where we would start, umm, raising funds, student clubs, you know, I think when I see change at the university it's been because students have pushed for it, right. So you get the students together and educate them on why this is important… research studies, putting the data together, when you show a need for it, I think the university is more likely to become more willing to spend the money, right? It's always about money. Or a state school, or publicly funded, it's always going to go down to who's gonna pay for these things? You know? Who's gonna be responsible for that. That, and then, business services I believe is the department that oversees, um, the VM and all the other types of products on campus so that could be a good place to start.” “Classrooms is a good way to go, also. If you talk to the professors, ask them for 5 min. Go to the class and talk to them, send it around, and see if they're going to sign the petition. Once you get a petition, maybe you could put one vending machine in place and go from there. See how it works and once it starts working then you can generate a profit for, then you could give that to the teacher and if that's what they wanna see then maybe that's what you can do. On a wide scale go and get signatures.”

#### Theme 1: VMs Are Convenient

The major reason students liked VMs was they were located all over campus, cheaply priced, opened 24 h, and thereby very convenient. Students expressed that VMs were fast and easy ways to quell their hunger on campus. Due to their class schedules and other time constraints that they often did not have time to visit restaurants to consume meals. VM fit into the busy lives of the students' and offered a variety of ways to purchase snack foods throughout campus.

#### Theme 2: VMs Are in Need of Improvement

Participants revealed their belief that VM required improvements both in the focus group discussions and in the surveys they completed. Participants noted that the variety and nutritional content of VM snacks were lacking and needed improvement. Many students suggested that VMs contained very unhealthy products and that they avoided VM because there were no healthier options. Some of the suggested healthier foods to place in VM included high protein foods, such as non-fat Greek yogurt and nuts, fresh fruits, whole grain options, fresh vegetables, healthy desserts, such as chocolate hummus and fruit sorbets.

#### Theme 3: Improving VM Content Will Be Challenging

The participants had many ideas for items they would like to see in VM and expressed a clear understanding that various VM products have differing shelf lives and potentially costs directly related to the healthfulness of the product. For example, fresh fruits and refrigerated items were among the preferred items but concerns about the cost of refrigerated machines, short shelf life, and low-profit margins were expressed. There was consensus among participants that healthy foods need to be well priced in order to be accessible to the student body and feasible for the university to make the switch to install healthy VMs. They discussed how if costs were not offset in some way improving the content might not be possible. In addition, they noted that healthier items may not sell as well due to a lack of knowledge among consumers about the expense of healthier options and recommended nutrition education to raise awareness on the importance of consuming healthier foods, including VM snacks. To increase the acceptability of improving VMs on campus, the students discussed showing authorities that there is a demand for healthier foods through a petition or a healthy VM trial run and collaborating with other organizations, and student groups.

## Discussion

The current study attempted to reveal college students' perceptions about items sold in VM and suggestions for improving these at a Hispanic serving institution. The most important points mentioned by participants in this study were that VM foods are convenient but unhealthy. They suggested including healthier options such as non-fat yogurt and nuts, fresh fruits and vegetables, whole grains, and healthy desserts. When referring to the implementation of these changes, they were concerned with the cost and suggested obtaining university funding, ensuring healthy products are being restocked appropriately, as well as gauging the success of a healthy VM with a trial run. Creating a health culture was said to be one of the topics that would create the most change, considering nutrition education and collaboration with other organizations on campus as the best approaches.

There are very limited qualitative studies assessing the college students' perceptions about items sold in VM, making it more difficult to compare the results from the present study to others. One of these studies was conducted among 43 undergraduate university students from the United Arab Emirates Ali et al. (17). This study also highlighted the importance of nutrition education as part of interventions to improve the quality of snacks purchased from VM has also been highlighted (17). These students expressed the need to improve the nutritional quality of snacks sold in these dispensers and recommended using nutrition education activities such as placing nutrition tips on or beside the VM and using active learning methods, such as competitions on nutrition knowledge (17). They also emphasized that students demanded nutrient-dense food products on campus, similar to our study. Another qualitative study in 40 high school students in Canada also reported the most students would like to have more healthy choices in their VM, although when available, they rarely purchased them (44). The cost of healthy items was reported as the largest barrier to purchasing them, similarly to the findings of the present study. Also, another barrier reported was the limited variety and unnoticeable placement of the snacks, which is also similar to the present study. Students also reported that they prefer healthy choices like fresh fruits, fresh vegetables, milk, and yogurt in vending machines, similar to our study results. The cost has also been reported by college students as one of the main limitations to purchase healthy snacks from VM, as evidenced in several cross-sectional studies (24, 45).

A few interventions have been implemented to improve the quality of the snacks offered in VM. A pilot study in a California university in the US evaluated a program to influence the point of purchase foods at a convenience store in one of the residence halls (20). Certain items were identified as healthy and sales of these healthy items increased after 5 weeks compared to baseline. Another study in seven US cities evaluated the impact of an intervention on two goals for the schools: to offer dessert and snack foods with <200 kcals per single-serving package and to eliminate 100% fruit juice and most sweetened beverages from VM (46). All intervention schools met their goals for snacks and beverages in VM, although the study did not evaluate if these changes resulted in dietary improvements in their students. Another trial in Canada among four high schools found that replacing non-healthy snacks with healthier snacks at a ratio of 50:50 led to lower sales and referred that the main barriers to purchasing the healthier options were price, value, and taste (44).

Nutrition education is key when implementing changes in VM and several trials have tested different educational strategies to improve the purchase of healthier items from VM. A trial in the Netherlands tested three strategies to increase consumption of healthier snacks, which were implemented in 13 high schools (47). These strategies were: improving the availability of healthy snacks in VM, labeling these products, and reducing their price. They showed that there were proportionally greater sales of the healthier snacks compared to non-healthy snacks with all these strategies, without affecting total snacks' sales. Another trial on a college campus tested these same strategies separately for improving healthy snack consumption (28). They found that when combining a higher availability of healthy options with promotional signs, sales of healthy items increased, while price reductions alone did not affect them. Similar, another study in a US college campus in Utah used labeling of healthy and unhealthy items from VM with a color-coded system (green for healthy, yellow for moderately healthy, and red for less leady items) and found that purchase of green items increased while red and yellow-coded items decreased (25).

Institutions should start with the initiative of offering more nutritious foods to their student body to improve health and wellbeing, but this type of initiative should include an institution-wide policy change. To our knowledge, only one university has implemented a policy to improve snacks sold in VM. The Ohio State University in the US implemented a policy in 2008 in which VM should be stocked with the following proportion of types of snacks: 28.5% of green (healthiest), 43% of yellow (less healthy), and 28.5% of red (non-healthy) items (16). However, evaluation of this policy through the sales of snacks in eight VM showed that it was highest for less healthy options (59%), and similar to the present study, the selection of these snacks was based on hunger (42%) and convenience (41%). At the worksite, the state of Delaware in the US implemented a policy among three state agencies to stock at least 75% of items in VM with healthy options (48). Although it took 7–19 weeks to implement the policy, total sales of snack items did not decrease, with some even increasing in sales.

Among the strengths of the study were the open peer-led discussion and the ease of participants in sharing their perceptions among a diverse group of students. Most had experienced purchasing foods from VM and therefore, had several recommendations to share. A limitation was the small sample size and the fact that those who chose to participate might have been biased toward a healthier lifestyle. Also, race/ethnicity information was not collected so participant remarks could not be stratified by this variable.

In conclusion, college students in this Hispanic serving institution want to see their snack environment improved and understand the obstacles to that change. They suggested changes to improve VM quality that will require both a university-wide policy on snacks and a holistic nutrition education intervention. The participants' suggestions for how to implement the changes reveal an understanding of business, communication, and consensus-building. Improving the snack environment at colleges may lead to higher consumption of healthy snacks which may lead to improving diet quality and this, in turn, could have long-lasting health benefits.

## Data Availability Statement

The raw data supporting the conclusions of this article will be made available by the authors, without undue reservation.

## Ethics Statement

The studies involving human participants were reviewed and approved by Florida International University Institutional Review Board. The patients/participants provided their written informed consent to participate in this study.

## Author Contributions

CM and CP designed the research. CM conducted the research. CM and CK analyzed the data and involved in interpreting the results and editing the manuscript. CM, CK, and CP wrote the paper. CP had primary responsibility for the final content. All authors read and approved the final manuscript.

## Funding

This study was funded in part by Florida International University.

## Conflict of Interest

The authors declare that the research was conducted in the absence of any commercial or financial relationships that could be construed as a potential conflict of interest.

## Publisher's Note

All claims expressed in this article are solely those of the authors and do not necessarily represent those of their affiliated organizations, or those of the publisher, the editors and the reviewers. Any product that may be evaluated in this article, or claim that may be made by its manufacturer, is not guaranteed or endorsed by the publisher.

## Abbreviations

US: United States
VM: vending machines
FIU: Florida International University.
